# Deubiquitinase USP47-stabilized splicing factor IK regulates the splicing of *ATM* pre-mRNA

**DOI:** 10.1038/s41420-020-0268-1

**Published:** 2020-05-04

**Authors:** Hye In Ka, Sunyi Lee, Sora Han, Ae Lee Jeong, Ji Young Park, Hyun Jeong Joo, Su Jung Soh, Doyeon Park, Young Yang

**Affiliations:** 1 Department of Biological Sciences, Sookmyung Women’s University, Seoul, 04310 Korea; 2 Drug Evaluation Group, R&D Center CJ HealthCare, Icheon, 04551 Korea; 3 Research Institute of Women’s Health, Sookmyung Women’s University, Seoul, 04310 Korea; 4 New Drug Development Center, Osong Medical Innovation Foundation, Osong, 28160 Korea

**Keywords:** Alternative splicing, RNA splicing

## Abstract

IK depletion leads to an aberrant mitotic entry because of chromosomal misalignment through the enhancement of Aurora B activity at the interphase. Here, we demonstrate that IK, a spliceosomal component, plays a crucial role in the proper splicing of the *ATM* pre-mRNA among other genes related with the DNA Damage Response (DDR). Intron 1 in the *ATM* pre-mRNA, having lengths <200 bp, was not spliced in the IK-depleted cells and led to a deficiency of the ATM protein. Subsequently, the IK depletion-induced ATM protein deficiency impaired the ability to repair the damaged DNA. Because the absence of SMU1 results in IK degradation, the mechanism underlying IK degradation was exploited. IK was ubiquitinated in the absence of SMU1 and then subjected to proteolysis through the 26S proteasome. To prevent the proteolytic degradation of IK, a deubiquitinating enzyme, USP47, directly interacted with IK and stabilized it through deubiquitination. Collectively, our results suggest that IK is required for proper splicing of the *ATM* pre-mRNA and USP47 contributes toward the stabilization of IK.

## Introduction

Spliceosomes are large complex molecular machines that remove introns from transcribed pre-mRNAs. Spliceosomes, which are associated with more than 150 accessory proteins, are formed from five different small nuclear ribonucleoproteins (snRNP)^1–5^. In the first step of splicing, the U1 snRNP recognizes the 5′-splice site (SS) region and U2 snRNP is recruited to the branch site (BS) region by binding the U2 auxiliary factor (U2AF). The Tri-U4/U6·U5 snRNP is recruited to the U1 and U2 assembly and the helicase Prp28 helps the displacement of the U1 snRNP at the 5′-splice site into the U6 snRNA. Simultaneously, B-specific proteins, including IK and SMU1, are recruited to the B complex^6,7^. This B complex is transformed into an activated spliceosome (B^act^) by unwinding of the U4/U6 RNA duplex triggered by the helicase Brr2. The remaining U6 snRNP interacts with the U2 snRNA. During the course of B activation, B-specific proteins, including IK and SMU1, are dissociated. Because IK and SMU1 have no orthologs in yeast, these two proteins are considered to act as regulatory proteins^8^. In addition to this splicing function of IK and SMU1, IK plays a role in the cell cycle regulation, as reported previously^9^. IK localizes to the spindle pole and colocalizes with MAD1 at the spindle poles. The depletion of IK leads to a failure of the spindle pole localization of MAD1^10^. The IK depletion is also able to disrupt the interaction between Aurora B kinase and protein phosphatase 2 (PP2A)^11^, leading to aberrant mitotic entry with chromosomal misalignment and chromosomal instability^12,13^.

Posttranslational modification of spliceosomal proteins allows the exact splicing of pre-mRNAs through dynamic regulation of protein interactions^14,15^. Presently, it is known that the U4/U6.U5 tri-snRNP complex of spliceosome is subjected to ubiquitination for dynamic activation of the spliceosome. The splicing factor, Prp8, a component of the U5 snRNP, is ubiquitinated within triple snRNPs yielding the stabilization of the U4/U6-U5 triple snRNP by repressing the U4/U6 unwinding^16^. PRP31, a component of the U4 snRNP, is modified with K63-linked ubiquitin chains by the PRP19 complex and is deubiquitinated by the ternary complex of USP15, SART3, and USP4. Thus, the ubiquitination and deubiquitination status of PRP31 regulates its interaction with the U5 snRNP component, PRP8, by stabilizing the U4/U6.U5 tri-snRNP complex^17^. Ubiquitin has seven lysine residues. K11- or K48-linked ubiquitinated proteins are the targets of degradation by 26S proteasome, whereas K63-linked ubiquitinated proteins mediate protein–protein interaction instead of proteolysis^18–20^. Until now, little is known about K11- or K48-linked ubiquitination of a spliceosomal component which might be relevant to regulation of splicing.

Activation of ataxia-telangiectasia mutated (ATM) kinase is an initial event in the DNA damage repair^21–23^. The activated ATM upon DNA damage leads to subsequent phosphorylation of downstream targets, including CHK1, CHK2, and p53^24–26^. These phosphorylations promptly initiate the recruitment of damage repair factors at DNA lesions to repair DNA double-strand breaks (DSBs). Previous studies showed that ATM and spliceosome are reciprocally regulated^27,28^. When RNA polymerase II (RNAPII) encounters DNA lesions, snRNPs to form spliceosome are displaced and followed by facilitation of R-loop formation, which results in non-canonical activation of ATM to repair damage. On the other hand, the activation of ATM also was known to regulate alternative pre-mRNA splicing^29^. However, the splicing mechanism of *ATM* pre-mRNA by spliceosome is not yet clearly understood.

In the present study, we observed that the depletion of the splicing factor IK leads to intron 1 retention in ATM, but not in ATR, indicating that IK stabilization is very important for the proper splicing of ATM. In addition, we demonstrate that the stability of spliceosomal protein IK is regulated by ubiqutination-mediated proteolysis. USP47, which belongs to the ubiquitin-specific protease (USP) family of deubiquitinating enzymes (DUBs), prevents the proteolysis of IK through deubiquitination.

## Materials and methods

### Cell culture

HeLa and HEK 293T cells obtained from the ATCC were maintained in Dulbecco’s modified Eagle’s medium (DMEM, Hyclone) supplemented with 10% heat-inactivated fetal bovine serum at 37 °C in a humidified 5% CO_2_ incubator, as described previously^13^. The cells were treated with the following DNA-damaging reagents: thymidine (Thy), mitomycin C (MMC), neocarzinostatin (NCS), camptothecin (CPT), etoposide (ETP), and hydroxyurea (HU). The cells were also treated with the protein synthesis inhibitor cycloheximide (CHX), the lysosomes inhibitor bafilomycin (Baf), the autophagy inhibitor wortmannin (Wor), and the proteasome inhibitor bortezomib (BTZ) or MG132.

### Plasmids

Full-length human IK and USP47 cDNAs were purchased from OriGene (OriGene Technologies, Inc., Rockville, MD) and cloned into pcDNA 3.1, pcDNA 3.0, and pCMV-tag-2B vectors. In the restoration assay, IK cloned into pCMV-tag-2B vector was used. The full-length mouse IK cDNA was purchased from Origene and cloned into pcDNA 3.1 vector. Plasmid transfection was performed using a polyethylenimine (PEI) solution, X-tremeGENE HP DNA Transfection Reagent, and jetPRIME (Polyplus) reagent, according to the manufacturer’s instructions.

### Antibodies

Primary antibodies used for immunoblotting and immunofluorescence analyses were as follows: rabbit polyclonal anti-IK (Santa Cruz, sc-1335485), rabbit polyclonal anti-IK (Bethyl Laboratories, A301-708A), mouse monoclonal anti-USP47 (Santa Cruz, sc-100633), mouse monoclonal anti-β-actin (Santa Cruz, sc-47778), rabbit monoclonal anti-pATM S1981 (Cell Signaling, #5883), rabbit monoclonal anti-ATM (Cell Signaling, #2873), rabbit polyclonal anti-pATR S428 (Cell Signaling, #2853), rabbit monoclonal ATR (Cell Signaling, #13934), rabbit monoclonal anti-pCHK1 S345 (Cell Signaling, #2348), rabbit polyclonal anti-pCHK1 S317 (Cell Signaling, #2344), rabbit polyclonal anti-pCHK2 T68 (Cell Signaling, #2661), mouse monoclonal anti-SMU1 (Santa Cruz, sc-100896), mouse monoclonal anti-Ub (Santa Cruz, sc-8017), mouse monoclonal anti-cleaved PARP (Cell Signaling, #9546), rabbit polyclonal anti-Cleaved Caspase-3 (Asp175) (Cell Signaling, #9661), rabbit monoclonal anti-Cleaved Caspase-9 (Asp315) (Cell Signaling, #20750), rabbit monoclonal anti-Mre11(Cell Signaling, #4847), rabbit polyclonal anti-pMre11(Ser676) (Cell Signaling, #4859), rabbit polyclonal anti-Rad50 (Cell Signaling, #3427), rabbit polyclonal anti-phospho p95 (Cell Signaling, #3001), rabbit monoclonal anti-p95 (Cell Signaling, #14956), rabbit monoclonal anti-phospho-Histone H2A.X (Ser139) (Cell Signaling, #9718), mouse monoclonal anti-HA (Santa Cruz, sc-7392), mouse monoclonal anti-FLAG (Sigma, F1804), mouse monoclonal anti-GFP (Santa Cruz, sc-9996), mouse monoclonal anti-SC-35 (Santa Cruz, sc-53518), and mouse monoclonal anti-BrdU (Cell Signaling, #5292) antibodies. The HRP-conjugated goat anti-mouse or anti-rabbit IgG (Fab) secondary antibodies were purchased from Enzo Life Sciences.

### RNAi

For RNA interference assays, IK siRNA duplexes were designed to repress IK (#1, 5′-CAAAGGUUGCAAGAUGUUU-3′; #2, 5′-CUACCAAGGAGUUGAUCAA-3′; #3, 5′-GCAUUCCAGUAUGGUAUCA-3′; #4, 5′-AGACCACACUGACCACAAA-3′; #5, 5′-AGCUGAGAUUGCCAGCAAA-3′) and were used at a concentration of 20 nM^10^. The SMU1 siRNA duplexes (5′-ACCACAGAAUGUUCAAAUA-3′) and USP47 siRNA duplexes (#1, 5′-GACUCUGAUAGUGUAGCAU-3′; #2, 5′-GCUCAGAUCCCUUUGGCUATT-3′; #3, 5′-GGCGUCAAGUCAACAUAUATT-3′) were also designed. The siRNAs were synthesized by Bioneer. For DUB siRNA screening, the Bioneer screening AccuTarget™ Human Ubiquitin siRNA set [SHS-0240] was used. The siRNAs were transfected into HeLa cells using Lipofectamine RNAiMax Transfection Reagent (Invitrogen) according to the manufacturer’s transfection protocol. For IK restoration assays, the cells were transfected with a human or mouse IK-expressing plasmid, and then with the IK siRNA 18 h after transfection with the IK plasmid. The cell lysates were prepared 48 h after transfection with the IK siRNA.

### Immunofluorescence

HeLa cells grown on coverslips were immediately permeabilized with 0.1% Triton X-100 in phosphate-buffered saline (PBS) for 3 min and were subsequently fixed with 4% paraformaldehyde in PBS for 10 min. The cells were then washed twice with PBS, permeabilized with 0.5% NP-40 in PBS for 5 min, and blocked with PBS-BT (3% BSA and 0.1% Triton X-100 in PBS) for 30 min at room temperature. The cells on the coverslips were subsequently incubated with primary and secondary antibodies diluted in PBS-BT for 1 h at RT. The nuclei of the fixed cells were stained with Hoechst 33258 or DAPI mounting medium. Images were acquired on an LSM‐700 Confocal Laser Scanning Microscope (Carl Zeiss) using a ×63 oil immersion objective lens and ZEN software (Nikon).

### Immunoblot analysis

For immunoblot analysis, cells were lysed in lysis buffer [50 mM Tris-HCl (pH 8.0), 150 mM NaCl, 1 mM EDTA, and 1% NP‐40, supplemented with a protease and phosphatase inhibitor mixture (Roche)]. Cell lysates were obtained by centrifugation for 15 min at 4 °C at 20,000 × *g*, and concentrations of the supernatants were quantified using the Pierce BCA Protein Assay Kit (Thermo Scientific). Total protein lysates were prepared using 5× SDS sample buffer and heating at 99 °C for 10 min. Proteins were separated electrophoretically on a SDS–polyacrylamide gel and transferred onto a 0.45‐μm pore size nitrocellulose membrane. The membrane was incubated overnight with antibodies containing 3% BSA in TBS‐T (150 mM NaCl, 20 mM Tris-HCl (pH 8.0), and 0.05% Tween‐20) at 4 °C, followed by incubation with HRP‐conjugated goat anti‐mouse or anti‐rabbit IgG (Fab) (Enzo Life Sciences) in 5% skim milk in TBS‐T at room temperature for 2 h. Proteins were visualized with ECL western blotting reagent and analyzed on a Fusion Solo‐S image analyzer (Vilber).

### Immunoprecipitation assay

Cells were lysed with lysis buffer [50 mM Tris-HCl (pH 8.0), 150 mM NaCl, 1 mM EDTA, and 1% NP‐40, supplemented with a protease and phosphatase inhibitor mixture (Roche)]. Cell lysates were obtained by centrifugation for 15 min at 4 °C at 20,000 × *g*. For endogenous protein immunoprecipitation, cell lysates were incubated with 3 μg of antibody for overnight followed by incubation with protein G agarose beads (Amicogen, 2010005) for 2 h at 4 °C. The immunocomplexes were then washed with lysis buffer for four times, and the immunocomplexes were separated by SDS–polyacrylamide gel and immunoblotting analysis was performed as described above.

### RNA immunoprecipitation

For RNA immunoprecipitation, HeLa cells were resuspended in nuclear isolation buffer (1.28 M sucrose, 40 mM Tris-HCl pH7.5, 20 mM MgCl_2_, 4% Triton X-100), and nuclear protein was extracted using the RIP buffer. The anti-IK antibody was mixed with HeLa nuclear extract in the RIP buffer, and then incubated overnight at 4 °C. Next, 30 μL of protein G-agarose beads was added to each binding reaction, and the samples were further incubated at RT for 3 h. The beads were subsequently washed five times with the RIP buffer, and RNA was extracted with TRIzol and reverse transcribed. The resulting cDNA was used as a template for PCR. The primers used for detecting ATM were designed to demonstrate that the detected signals were due to the RNA specifically binding to IK.

### Subcellular fractionation

Cell fractionation was performed using the PARIS kit (Ambion), according to the manufacturer’s instructions. The total isolated RNA was reverse transcribed using reverse transcriptase (Fermentas). The resulting cDNA was used as a template for PCR with the indicated primer sets. The values were normalized to those of GAPDH.

### ATM mini-gene splicing assay

The 262-bp human ATM mini-gene construct was cloned into the pEGFP-N2 vector. The ATM fragment containing exon 1 with a 72-bp added start codon, 79-bp of intron 1 (containing the 5′-GU-A-AG-3′, splicing recognition site), and 111-bp exon 2, flanked by the engineered *EcoR*I and *BamH*I restriction sites, was amplified from the cDNA extracted from the IK-depleted cells. The mini-gene construct (0.5 μg) was transfected with 2 μL of HP DNA transfection reagent (Roche), 24 h after siRNA transfection. After an additional 24 h incubation, protein was extracted using the lysis buffer and immunoblotted with an anti-GFP antibody, and the expression of green fluorescence protein (GFP) was observed by fluorescence microscopy.

### Metaphase spreads

The cells were arrested by treating with colcemid (Sigma-Aldrich) at a concentration of 0.2 μg/mL for 90 min, then harvested by incubating for 15 min at 37 °C in 0.075 M KCl, and subsequently fixed in a freshly prepared methanol:acetic acid (3:1 v/v) solution. Metaphase spreads were prepared by dropping the cell suspension onto slides pre-wetted with ddH_2_0. The slides were dried at 42 °C for 60 min before staining with DAPI mounting medium (Sigma-Aldrich) in Gurr Buffer for 3 min. After rinsing with fresh Gurr Buffer followed by rinsing with distilled water, the slides were fully dried and then monitored under a Zeiss microscope^30^.

### RT-PCR

For RNA extraction, 500 μL of the TRIzol reagent was directly added to cell culture plates, and the suspension was harvested and mixed with 100 μL of chloroform. After centrifugation, 200 μL of the clear supernatant was mixed with 200 μL of 100% isopropanol and centrifuged at 12,225 × *g* for 15 min at 4 °C. The pellet was resuspended in 30 μL of DNase/RNase free DW (Invitrogen), and the concentration of total RNA was measured using an Epoch microplate. The RNA samples (3 μg) were reverse transcribed using random hexamer primers. For quantification of band intensities, images from different sets of experiments were analyzed using the Image J software. The primer sequences used for RT-PCR analysis are available in Supplementary Table S1.

### Cell viability assay

To assess the effects of DNA damage inducers on the viability of IK-depleted cells, HeLa cells were transfected with siIK #1 for 24 h, and the cells were plated in 48-well plates. CPT and ETP (10 μM) were added to the cells 48 h after transfection, and the kinetics of the cells was monitored using an IncuCyte instrument (ESSEN BioSCIENCE).

### Annexin V staining

To assess the cell death, the transfected HeLa cells were seeded onto 6-well plates, treated with trypsin-EDTA, centrifuged, and resuspended in PBS. Thereafter, the cell suspensions were incubated at room temperature with PI/Annexin V for 15 min and analyzed using BD FACS Canto II. All the antibodies were purchased from eBioscience (eBioscience Inc. San Diego, CA).

### Statistical analysis

Values are presented as the means ± standard deviation (SD). Multiple comparisons within groups were performed by one-way analysis of variance (ANOVA), and differences between the means of individual groups were evaluated using Student’s *t*-test. A value of *p* < 0.05 was considered as the threshold for significant differences (*p < 0.05, **p < 0.01, ***p < 0.001).

## Results

### IK depletion results in abnormal fragmentation of chromosomes

**Fig. 1:**
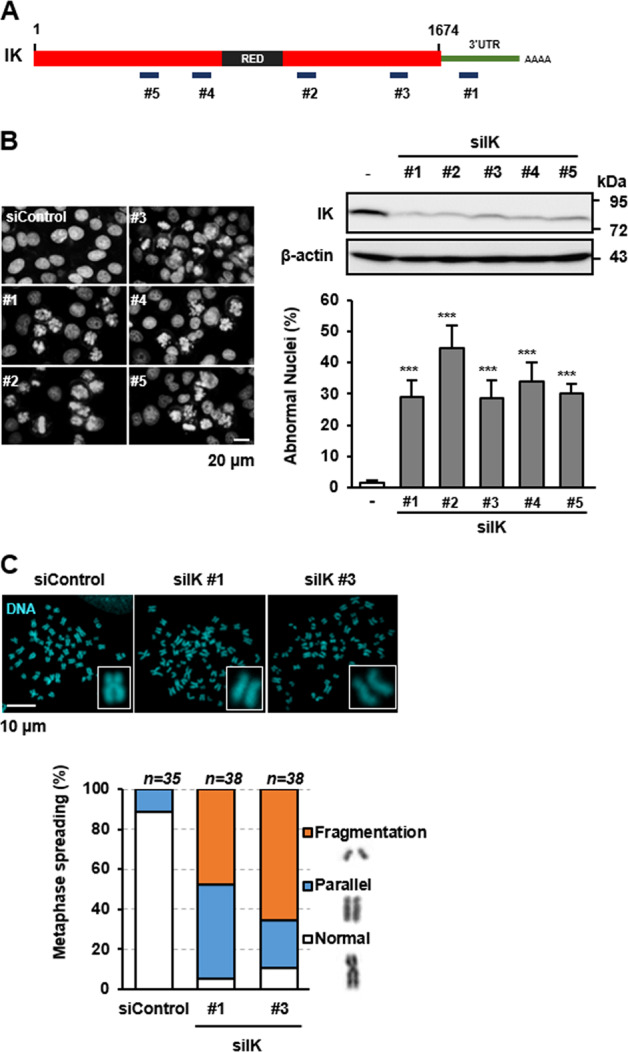
IK depletion results in abnormal fragmentation of chromosomes. **a** Schematic representation of the IK siRNA target sites in IK. **b** Cells transfected with five different siIK constructs were stained with Hoechst 33258 after transfection for 48 h. Changes in nuclear morphology were examined by confocal laser microscopy, and the level of IK was evaluated. Quantification was performed in quintuplicate (*n* > 100); the graph shows the percentage of abnormal nuclei in each case. **c** Cells transfected with siIK #1 or siIK #3 were used to prepare the metaphase spreads. Representative images of metaphase spreads from the IK-depleted cells obtained by confocal laser microscopy were examined, and the results of quantification of the chromosomal shapes were graphed. ***p < 0.001.

We have previously shown that IK depletion causes a marked increase in the proportion of mitotic cells showing misaligned chromosomes because of the increase in phosphorylation of Aurora A kinase, Aurora B kinase, and PLK1^11,13^. In the present study, we classified the various aberrant chromosomal structures induced by the IK depletion. To this end, five different siRNAs targeting various regions of IK, including the coding and 3′-untranslated regions (UTRs), were designed (Fig. 1a). All the five IK siRNAs successfully reduced the IK expression in HeLa cells and caused significant nuclear abnormalities (Fig. 1b). Because IK siRNA #1 targeted the 3′-UTR, we used it for subsequent experiments including a rescue experiment. To classify the aberrant chromosomal structures, we examined the metaphase chromosome spreads after transfection with siIK #1 or siIK #3. The loss of IK resulted in various atypical chromosome shapes, including a parallel shape indicating cohesion defects and a fragmented shape indicating an impaired DNA repair system (Fig. 1c). Together, these results revealed that IK plays a crucial role in genome stability and stable cell viability in human cells.

### IK depletion inactivates the DNA damage-induced ATM signaling pathway via decrease in the ATM level

**Fig. 2:**
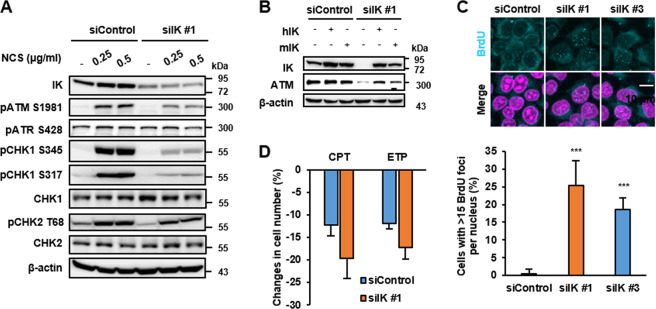
IK depletion inactivates the DNA damage-induced ATM signaling pathway via decrease in the ATM level. **a** Cells transfected with siIK #1 for 47 h were treated with neocarzinostatin (NCS) at the indicated concentrations for an additional 1 h, and the levels of IK, pATM S1981, pATR S428, CHK1, CHK2, pCHK1 S345, pCHK1 S317, and pCHK2 T68 were examined. **b** Cells transfected with human IK or mouse IK for 18 h were transfected with siIK #1 for an additional 48 h, and the levels of IK and ATM were examined. **c** Cells transfected with siIK #1 for 24 h were treated with BrdU at 100 μM for an additional 24 h and stained with an anti-BrdU antibody. Representative images obtained by confocal laser microscopy were examined. The percentages of cells exhibiting more than 15 BrdU foci per nucleus (*n* > 162) are graphed. ***p < 0.001. **d** Cells transfected with siIK #1 for 48 h were treated with 10 μM camptothecin (CPT) or etoposide (ETP) for an additional 24 h. The cell growth was monitored for an additional 24 h using an IncuCyte live-cell imaging system. *n* = 3.

Because IK depletion-induced chromosome fragmentation, we inferred that IK might be associated with the DNA damage repair system. To determine whether IK affects the DNA damage repair pathway under genotoxic stress, we treated the IK-depleted HeLa cells with neocarzinostatin (NCS), which causes DNA DSBs. In response to NCS treatment, IK-depleted cells showed a weak increase protein levels in ATM phosphorylation at S1981, CHK1 phosphorylation at S345, S317, and CHK2 phosphorylation at T68, but the levels of ATR protein and phosphorylation were not affected (Fig. 2a). To further confirm this result, the IK-depleted cells were also treated with other DNA damage inducers including thymidine (Thy), mitomycin C (MMC), camptothecin (CPT), etoposide (ETP), and hydroxyurea (HU), and showed a similar pattern with NCS treatment (Supplemental Fig. S1a, b). In addition, IK-depleted cells showed low percent of co-localization between phosphorylated ATM and phosphorylation of the histone variant H2AX (γH2AX) foci, which is formed when double-strand breaks appear upon HU treatment (Supplemental Fig. S1c, d), which is likely to due to lack of ATM amount. Next, to completely exclude the possibility that the decrease in ATM was due to an off-target effect of the IK siRNA, we transfected full-length human or mouse IKs to IK-depleted cells, and the ATM protein level was almost restored in the IK-knockdown cells (Fig. 2b). Because IK depletion causes a decrease in the ATM protein levels, we hypothesized that the IK-depleted cells would exhibit an impairment of the ATM-mediated DNA damage repair. To examine this hypothesis, BrdU incorporation assay was performed to measure the frequency of ssDNA breaks in the IK-depleted cells. The IK-depleted cells showed a dramatic increase in the formation of BrdU foci (Fig. 2c). However, it is possible that the increase in BrdU incorporation would be due to DNA damage as well as checkpoint defect. To further prove this possibility, the IK-depleted cells were treated with CPT and ETP and showed significantly reduction in cell number (Fig. 2d).The DNA damage-inducing drugs treatment may affect the IK-depleted cells highly susceptible to decrease in cell proliferation. Collectively, these results indicate that IK is associated with the DNA damage repair system through the regulation of endogenous ATM protein levels.

### IK depletion decreases the spliceosomal excision of intron 1 in the *ATM* pre-mRNA

**Fig. 3:**
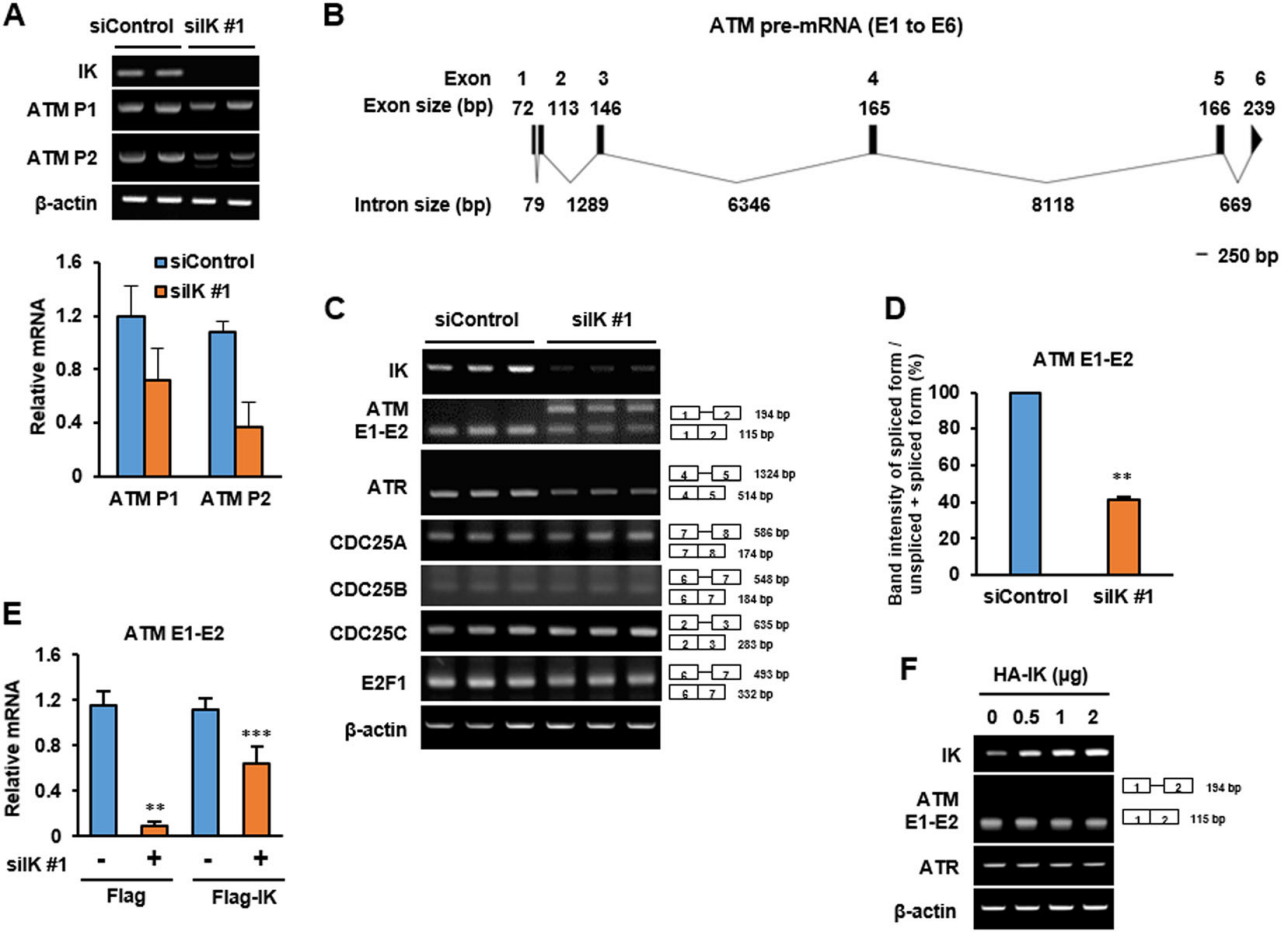
IK depletion decreases the spliceosomal excision of intron 1 in the *ATM* pre-mRNA. **a** Cells were transfected with siIK #1 for 48 h, and the levels of *ATM* mRNA were determined. The intensity of each band was graphed. *n* = 3. **b** A schematic view of the ATM pre-mRNA (E1 to E6). Scale bar = 250 bp. **c** Cells were transfected with siIK #1 for 48 h, and the levels of *ATM*, *ATR*, *CDC25A/B/C*, and *E2F1* mRNA were determined. **d** A graph of the percentage spliced in (PSI), which correspond to (Intensity of the spliced form PCR band)/(intensity of the unspliced + intensity of the spliced form PCR bands) from ATM E1–E2 in **c**. **e** Cells overexpressed with FLAG-IK for 18 h were transfected with siIK #1 for an additional 48 h, and the relative *ATM* mRNA (E1–E2) were determined using qRT-PCR. **f** Cells were transfected with HA-IK for 24 h, and the levels of IK, *ATM E1-E2*, and *ATR* mRNA were determined.

Because IK functions as a splicing factor, it was determined whether the decrease of ATM protein levels in the IK-depleted cells was related to the aberrant function in spliceosomes. Thus, first, the levels of mRNA were examined using exon–exon specific primers designed based on different *ATM* exon regions and the level of *ATM* mRNA was found to be decreased in the IK-depleted cells (Fig. 3a). Because previous report showed IK was related in splicing of short intron^8^, we also checked the intron sizes in ATM pre-mRNA using the human NCBI transcript reference sequences (Refseq: NM_000051) and displayed as a schematic view in ATM E1-E6 (Fig. 3b). We designed only primer targeting exon1 and exon 2 region which is only short length in ATM pre-mRNA and performed RT-PCR. The intron 1 retention between exon 1 and exon 2 in the *ATM* pre-mRNA was markedly increased in the IK-depleted cells (Fig. 3c), showing a ratio of ~40% spliced intensity in ATM pre-mRNA (Fig. 3d). However, *ATR* splicing, which is responsible for the single-strand DDR, was processed normally. In addition, *CDC25A*, *CDC25B*, and *CDC25C*, which are inhibited by the ATM-mediated pathway^31^, and the *E2F1* transcription factor, which increases the *ATM* pre-mRNA levels^32^, were also normally processed without any intron retention. From these result, we confirmed that IK is a critical splicing factor involved in the processing of *ATM* pre-mRNA. In addition, the intron retention of *ATM* pre-mRNA splicing was slightly rescued when IK was re-expressed using a siIK-resistant expression plasmid (Fig. 3e). There was no significant effect in *ATM* splicing at exons 1–2 upon IK overexpression (Fig. 3f). Together, these findings suggest that IK depletion decreases the splicing of ATM pre-mRNA especially between exon1 and 2.

### IK depletion causes intron retention of *ATM* pre-mRNA and IK directly binds to the *ATM* pre-mRNA

**Fig. 4:**
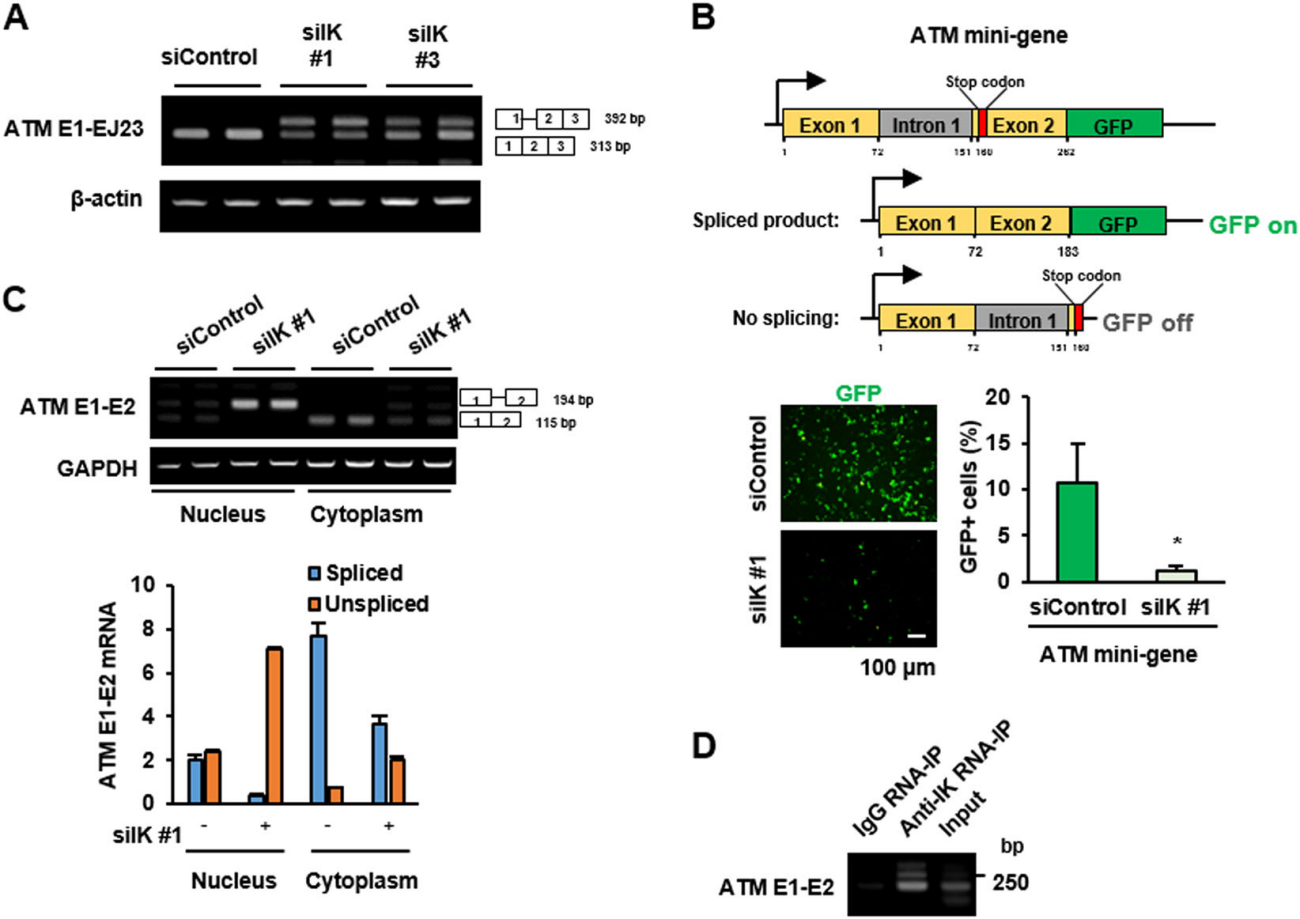
IK depletion causes intron retention of ATM pre-mRNA and IK directly binds to the *ATM* pre-mRNA. **a** Cells were transfected with siIK #1 or siIK #3 for 48 h, and the levels of *ATM* E1-EJ23 mRNA were determined. **b** Cells transfected with siIK #1 for 24 h were transfected using the GFP-*ATM*-mini-gene construct for an additional 24 h, and the levels of IK and GFP were examined. Representative images of green fluorescent protein-expressing cells obtained using fluorescence microscopy were examined, and the intensity of green fluorescence was quantified. *n* = 3. *p < 0.05. **c** Cells were transfected with siIK #1 for 48 h, and cytoplasmic RNA and nuclear RNA were extracted separately. The levels of *ATM* mRNA were examined, and the intensity of each band was graphed. *p < 0.05. **d** HEK-293T cells were immunoprecipitated with anti-IK antibodies, and the levels of *ATM* mRNA were determined.

Next, it was determined whether exon skipping is a possible event in the IK-depleted cells and the region between exons 1 and 2 was examined. In IK-depleted cells, intron 1 retention was observed without exon skipping (Fig. 4a). To further validate the intron 1 retention, we constructed an *ATM* mini-gene containing exon 1–intron 1–exon 2 positioned upstream of the GFP-coding DNA (Fig. 4b). The IK-depleted cells failed to remove the intron 1. Thus, the level of GFP expression was markedly decreased, owing to a premature stop codon arising from a reading frame shift resulting from intron 1 retention in the absence of IK (Fig. 4b). Furthermore, we examined the RNA levels of *ATM* in the cytoplasmic and nuclear fractions of IK-depleted cells to determine whether intron retention-*ATM* transcripts was exported to the cytoplasm. Most of the intron 1-retaining forms of *ATM* transcript accumulated in the nuclear fraction, without being exported to the cytoplasm (Fig. 4c). Next, to confirm whether the *ATM* transcript localizes at the spliceosome containing IK, the co-presence of *ATM* transcript and IK protein in the nuclear extracts was examined. For confirmation of their co-presence, we precipitated nuclear extracts of HeLa cells with an anti-IK antibody and used the precipitates to amplify the *ATM* E1-E2 pre-mRNA primer. The amplified *ATM* bands were observed in the anti-IK precipitates but not in the control IgG precipitates (Fig. 4d).

### IK is degraded by the proteasome complex in the absence of SMU1

**Fig. 5:**
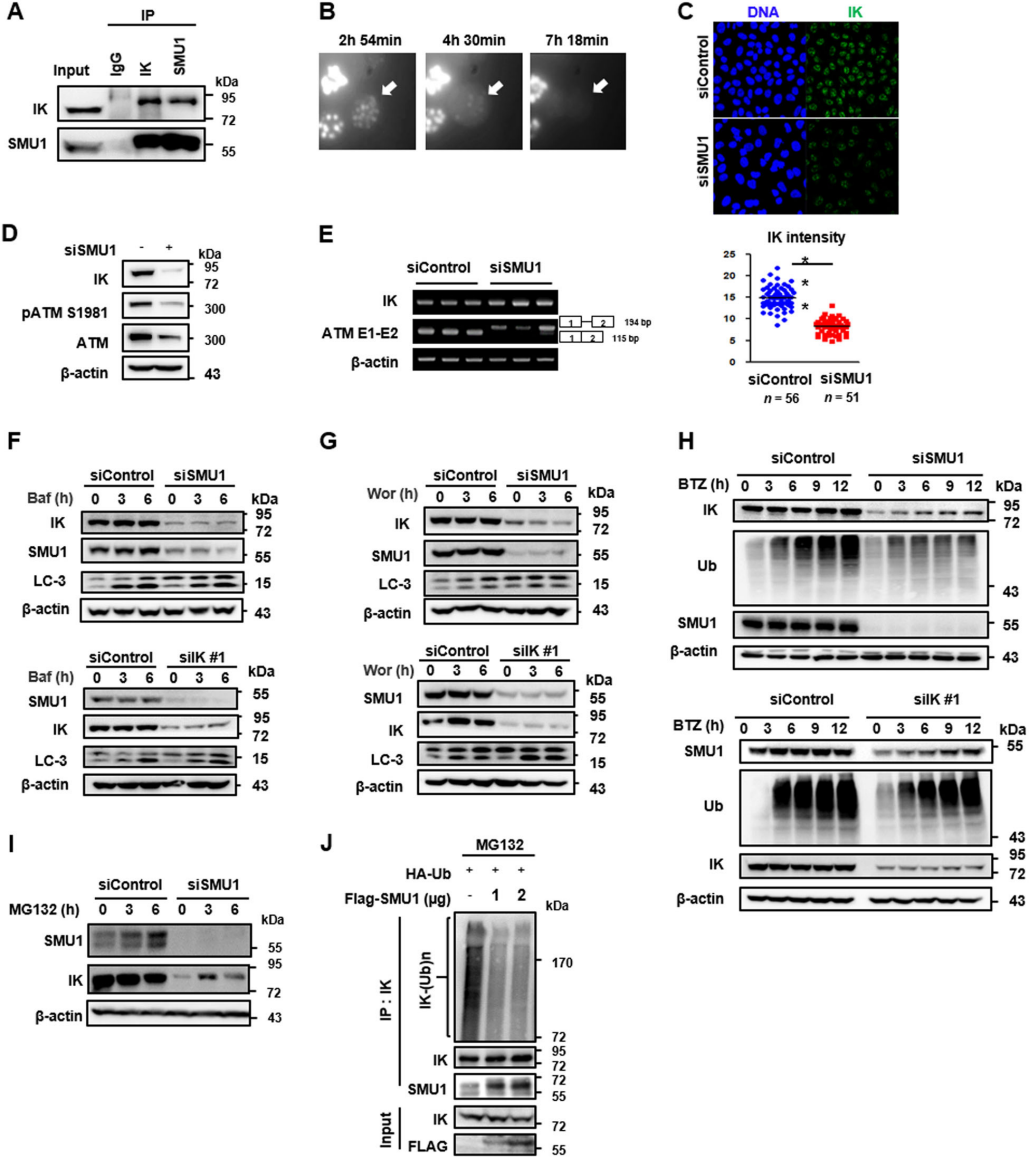
IK is degraded by the proteasome complex in the absence of SMU1. **a** Cells were immunoprecipitated with anti-IK and anti-SMU1 antibodies, and the levels of IK and SMU1 were examined. **b** Mitotic division of HeLa cells expressing GFP-tagged IK were recorded using time-lapse microscopy. Time from round-up is indicated. IK localized with nuclear speckle was marked with an arrow. **c** Cells transfected with siSMU1 for 48 h were stained with an anti-IK antibody. Representative images obtained using confocal laser microscopy were examined, and the intensity of IK was quantified using the ZEN software package and graphed with GraphPad Prism 5. ***p < 0.001. **d** Cells were transfected with siSMU1 for 48 h, and the levels of IK, pATM S1981, and ATM were examined. **e** Cells were transfected with siSMU1 for 48 h, and the levels of *ATM* mRNA were determined. **f** Cells were transfected with siSMU1 or siIK #1 for 42 h and treated with Bafilomycin at 100 nM for indicated times. LC3 is used as positive control of Autophagy. **g** Cells were transfected with siSMU1 or siIK #1 for 42 h and treated with Wortmannin at 5 µM for indicated times. LC3 is used as positive control of Autophagy. **h** Cells were transfected with siSMU1 or siIK #1 for 42 h and treated with Bortezomib at 200 nM for indicated times. **i** Cells were transfected with siSMU1 for 42 h and treated with 5 μM MG132, and the levels of SMU1 and IK were examined. **j** HEK-293T cells transfected with HA-Ub and FLAG-SMU1 for 24 h were treated with MG132 at 5 μM for 5 h and subjected to immunoprecipitation with an anti-IK antibody. The levels of ubiquitin, IK, and SMU1 were examined. *p < 0.05, **p < 0.01, ***p < 0.001.

The interaction between SMU1 and IK is essential for their spliceosomal functions and depletion of SMU1 or IK causes the changes in the expression level and alternative splicing profiles of several genes with intron specific effect^33^. We confirmed an endogenous interaction of IK with SMU1 using a pull-down assay (Fig. 5a). Next, we have examined the localization of IK within nuclear speckles where splicing factors are stored when not used in splicing by co-staining with SC35. The IK was co-stained with SC35 (Supplementary Fig. S2) and disappeared during mitosis (Fig. 5b). When SMU1 was depleted, the level of the IK was reduced (Fig. 5c). Thus, SMU1 depletion resulted in a decrease in the ATM total and phosphorylation protein level as well as in the IK expression level (Fig. 5d). To investigate whether SMU1 depletion decreases the efficiency of *ATM* pre-mRNA splicing as observed in the case of IK depletion, the splicing of the *ATM* pre-mRNA was examined in the SMU1-depleted cells. The loss of SMU1 increased intron retention between exon 1 and exon 2 similar to that observed with IK depletion (Fig. 5e), suggesting that *ATM* pre-mRNA splicing is regulated by IK which is stabilized by SMU1 binding. Next, to determine how SMU1 affects the stability of the IK protein, the SMU1-depleted cells were treated with bafilomycin (Baf) to inhibit the lysosomes (Fig. 5f), wortmannin (Wor) to inhibit the autophagy (Fig. 5g), and bortezomib (BTZ) to inhibit the proteasome (Fig. 5h). When treated with BTZ, degradation of IK was only inhibited in SMU1-depleted cells. Moreover, we observed that another representative proteasome inhibitor, MG132 treatment also inhibited the IK degradation induced by SMU1 depletion (Fig. 5i). To further confirm this observation, we examined the ubiquitination of IK in cells overexpressing SMU1. The overexpression of SMU1 inhibited the ubiquitination of IK (Fig. 5j), indicating that SMU1 is required for the stabilization of IK. Taken together, these data shows IK is degraded by the proteasome complex in the absence of SMU1.

### USP47 is a candidate deubiquitinase for IK

**Fig. 6:**
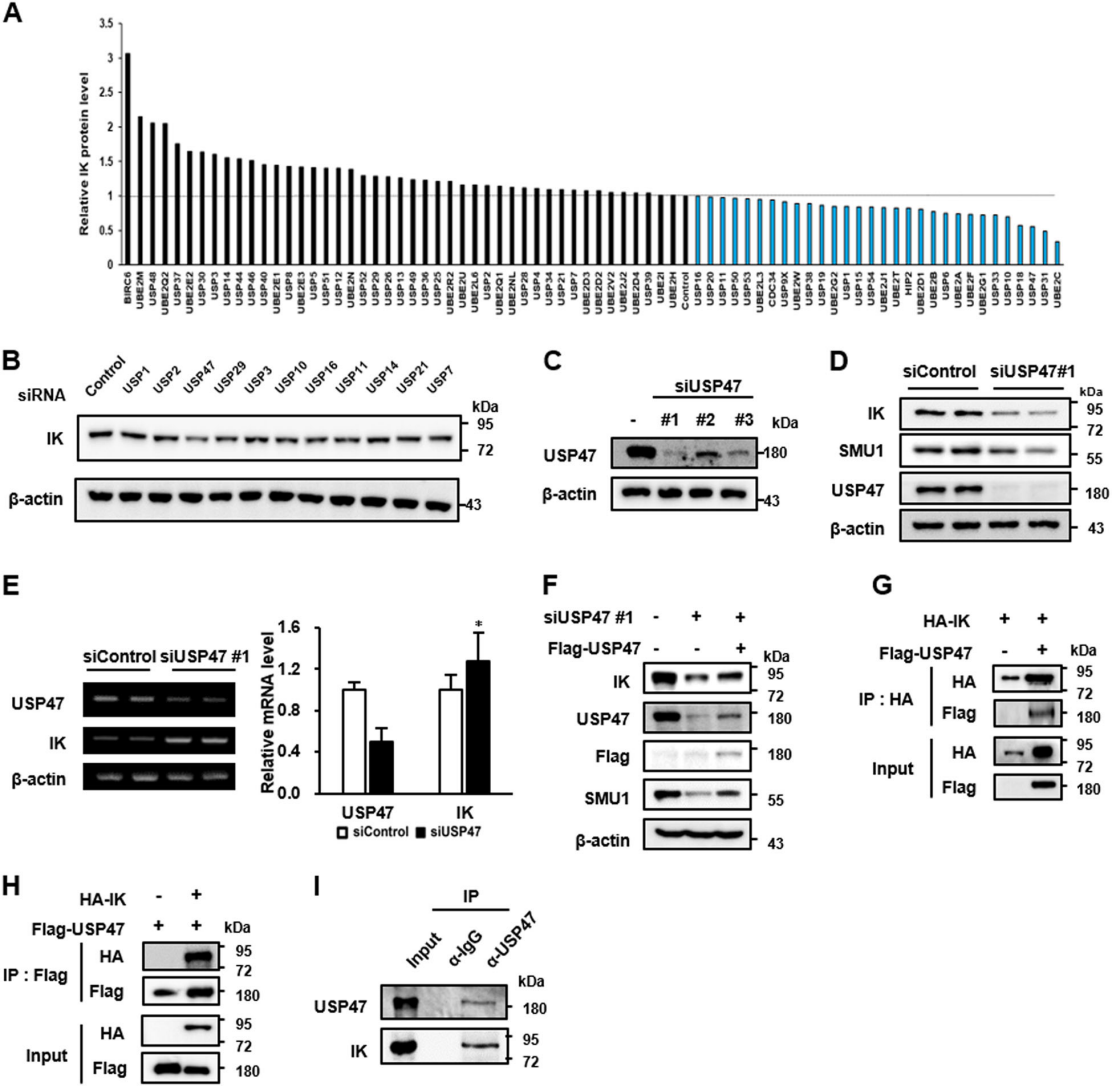
USP47 is a candidate deubiquitinase for IK. **a** HeLa cells were transfected for 48 h with individual siRNAs from a pool of 76 siRNA targeting deubiquitinases (DUBs). Next, the level of endogenous IK was measured by western blotting and protein bands were quantified using the Image J software. **b** Cells were transfected with 11 DUB siRNAs involved in DNA Damage Response (DDR), and the level of IK was examined. **c** Cells were transfected with three different siUSP47s for 48 h, and the levels of USP47 were examined. **d** Cells were transfected with siUSP47#1 for 48 h, and the levels of IK, SMU1, and USP47 were examined. **e** Cells were transfected with siUSP47#1 for 48 h, and the levels of *IK* mRNA were determined. The relative ratios of mRNA were normalized to those of β-actin and graphed. *p < 0.05. **f** Cells transfected with siUSP47 #1 for 24 h were transfected with Flag-USP47 for an additional 24 h, and the levels of IK and SMU1 were determined. **g**, **h** HEK-293T cells transfected with HA-IK or/and Flag-USP47 were immunoprecipitated with an anti-HA or anti-Flag antibody, and the levels of HA and FLAG were examined. **i** HeLa cells were immunoprecipitated with anti-USP47, and the levels of IK and USP47 were examined.

Because IK is regulated by a ubiquitin-dependent mechanism, we further investigated as to which deubiquitinating enzymes (DUBs) are able to stabilize IK. To identify the DUBs that could regulate the IK stability, the IK protein levels were determined after the treatment of siRNAs targeting each of the 76 DUBs (Fig. 6a; Supplementary Fig. S3). Among the 76 DUBs, 11 that are known to associate with DDR were separately examined because IK may play a role in the DNA damage repair (Fig. 6b). The USP47 depletion consistently decreased the endogenous levels of the IK protein. Next, USP47 was depleted with three different siRNAs targeting different regions of the USP47 (Fig. 6c). Among three siRNAs, siRNA#1 targeting the 3′-UTR was most effective and was, therefore, used in subsequent experiments. The USP47-depleted cells showed a decrease in the levels of both IK and SMU1 (Fig. 6d) without any decrease in the IK mRNA levels (Fig. 6e). Moreover, the USP47 overexpression rescued the levels of IK and SMU1 in the USP47-depleted cells (Fig. 6f). Next, to examine whether USP47 directly binds to IK, cell lysates expressing HA-IK and Flag-USP47 were used in a pull-down assay with anti-HA or -Flag antibodies. USP47 and IK were observed in both the immunoprecipitates (Fig. 6g, h). In addition, cell lysates obtained without the overexpression of HA-IK and Flag-USP47 were immunoprecipitated with the anti-USP47 monoclonal antibody. The endogenous IK was also included in the immunoprecipitates (Fig. 6i). Taken together, these results imply that USP47 should be a deubiquitinase responsible for the regulation of the endogenous IK.

### USP47 stabilizes IK through deubiquitination

**Fig. 7:**
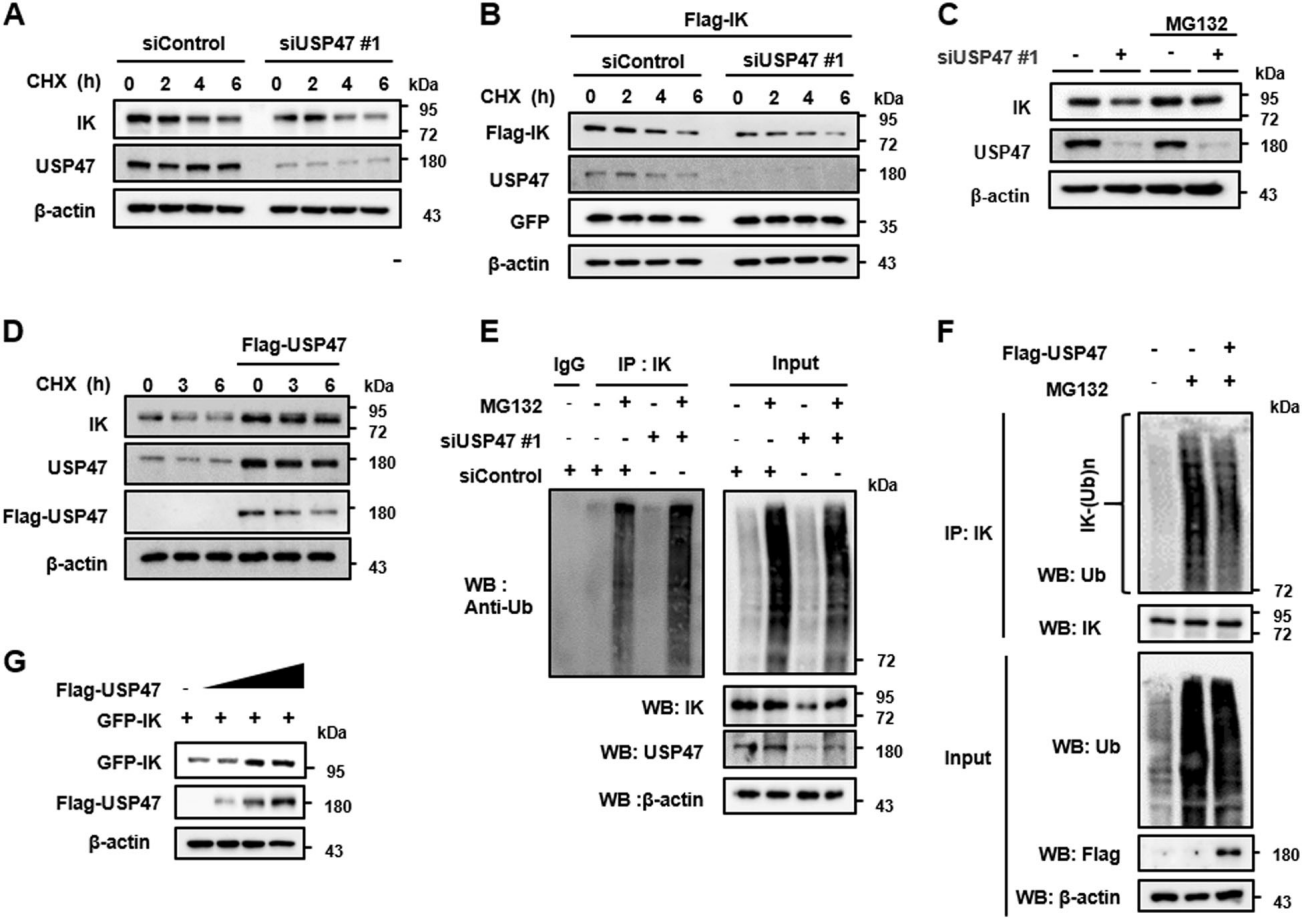
USP47 stabilizes IK through deubiquitination. **a** Cells were transfected with USP47#1 and treated with 40 μg/mL of cycloheximide (CHX) for the indicated times to determine the levels of IK. **b** Cells were transfected with siUSP47#1 for 24 h and incubated with Flag-IK for an additional 24 h, and the levels of IK were examined. Green-fluorescent protein (GFP) was used as a transfection control. **c** Cells were transfected with siUSP47#1 for 48 h and treated with 10 μM MG132 for an additional 6 h, and the levels of IK were detected. **d** Cells transfected with Flag-USP47 for 24 h were treated with 40 μg/mL of CHX for the indicated times and the levels of endogenous IK were examined. **e** Cells were transfected with siUSP47#1 for 42 h and treated with 10 μM MG132 for 6 h. Cells were lysed and immunoprecipitated with an anti-IK antibody, followed by immunoblotting using an anti-Ub antibody. **f** Cells were transfected with Flag-USP47 and treated with 10 μM MG132 for 6 h. Cells were lysed and immunoprecipitated using an anti-IK antibody, followed by immunoblotting using an anti-Ub antibody. **g** Cells were transfected with a GFP-IK and increasing amounts of Flag-USP47, and the levels of GFP-IK and Flag-USP47 were examined.

To further confirm the role of USP47 as a deubiquitinase of IK, the USP47-depleted cells were treated with an inhibitor of protein translation cycloheximide (CHX), to examine the IK stability. The USP47-depleted cells showed markedly reduced half-life of the endogenous IK compared to that in the non-depleted cells (Fig. 7a). Subsequently, the USP47-depleted cells and non-depleted cells were transfected with Flag-IK, and then treated with CHX. The USP47-depleted cells showed consistently reduced exogenous levels of IK (Fig. 7b). Next, to examine whether the degradation of IK in the USP47-depleted cells was mediated through the proteasomal pathway, the USP47-depleted cells were treated with the proteasome inhibitor, MG132. The IK degradation was impaired in the USP47-depleted cells treated with MG132 (Fig. 7c). In addition, the overexpression of USP47 increased the half-life of IK (Fig. 7d). To provide more direct evidence for the deubiquitination of IK by USP47, IK was immunoprecipitated from the lysates of USP47-depleted cells after MG132 treatment and then the level of ubiquitination was determined. The level of IK ubiquitination in the MG132-treated cells was greatly increased compared to that in the non-treated USP47-depleted cells (Fig. 7e). Moreover, forced expression of USP47 decreased the level of ubiquitinated-IK even after the MG132 treatment (Fig. 7f) and the expression of USP47 increased the level of IK in a dose-dependent manner (Fig. 7g).

### USP47 depletion decreases the level of ATM through IK degradation

**Fig. 8:**
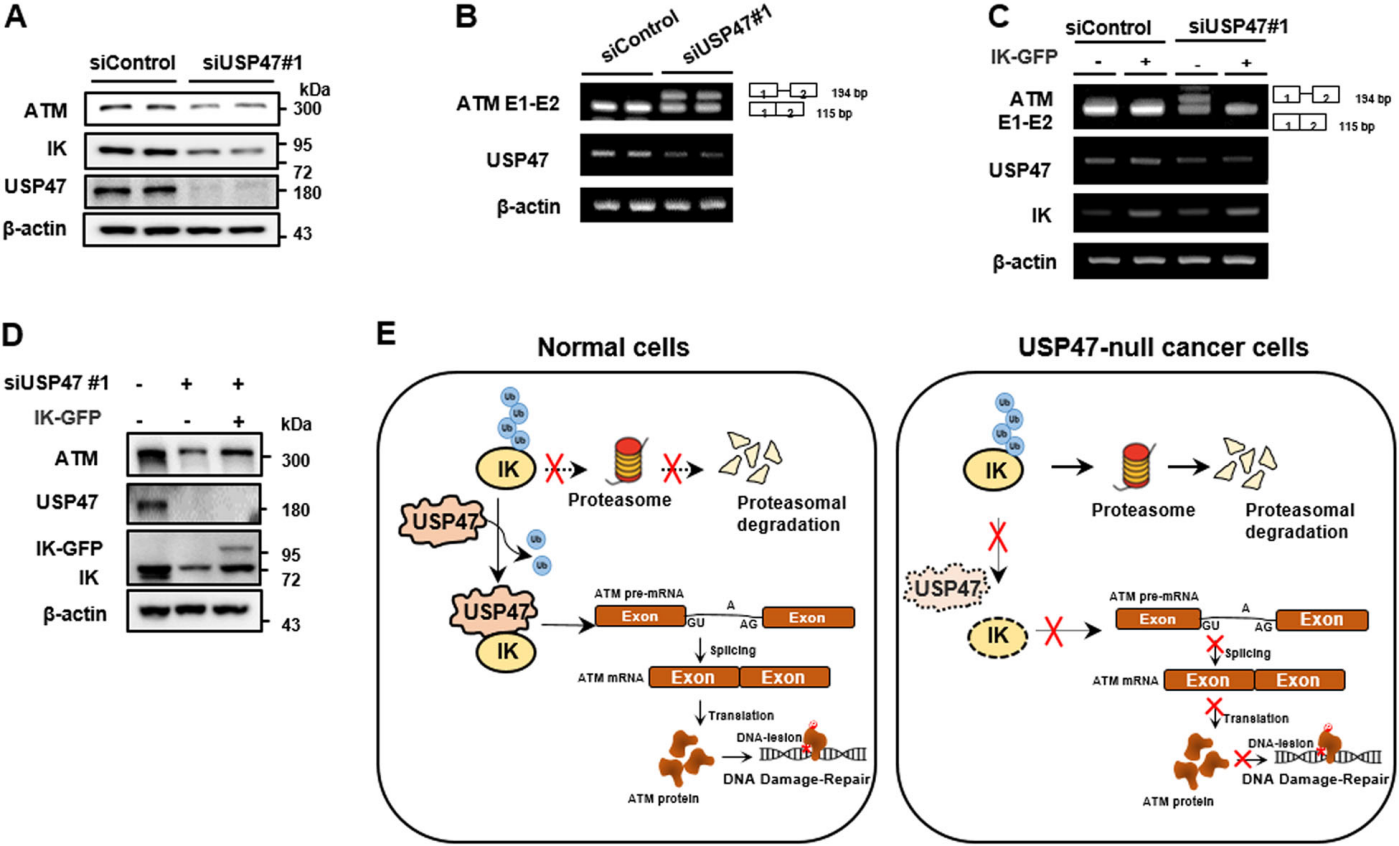
USP47 depletion decreases the level of ATM through IK degradation. **a** Cells were transfected with siUSP47#1 and the levels of ATM, IK, and USP47 were examined. **b** Cells were transfected with siUSP47#1 for 48 h, and the levels of *ATM* E1-E2 mRNAs were determined. **c** To restoration assay, cells transfected with siUSP47#1 for 24 h were transfected with IK-GFP for an additional 24 h, and the levels of *ATM* E1-E2 mRNAs were examined. **d** Cells transfected with siUSP47#1 for 24 h were transfected with IK-GFP for an additional 24 h, and the levels of ATM and IK were examined. **e** Schematic for the proposed function and relationship between IK and USP47.

Because IK is associated with the proper splicing of *ATM* pre-mRNA, the ATM levels were examined in the USP47-depleted cells. Expectedly, the USP47-depleted cells which have low levels of IK showed lower levels of ATM compared to that in the non-depleted cells (Fig. 8a). Moreover, the ratio between intron retention/spliced isoforms of *ATM* transcript at the junction of exons 1–2 was increased (Fig. 8b). To examine whether the aberrant splicing of *ATM* transcript at exons 1–2 could be rescued by restoration of IK, the USP47-depleted cells were transfected with IK-GFP, and successful ATM pre-mRNA splicing at exons 1–2 was observed (Fig. 8c). Moreover, the overexpression of IK partially rescued the low level of ATM protein upon USP47 depletion (Fig. 8d). Besides, to examine whether the USP47-depleted cells have an impairment of the ATM-mediated DDR, the USP47-depleted cells were treated with HU for 24 h (Supplementary Fig. S4a). Similar to the result observed in the IK-depleted cells (Supplementary Fig. S1b), the USP47-depleted cells showed a slight increase in ATM phosphorylation upon treatment of HU. It seems to be due to the decrease in the ATM protein levels upon IK depletion. Because the failure of ATM-mediated DDR increases apoptosis, the USP47-depleted cells treated with HU were collected undergoing apoptosis in a living context. The HU-treated USP47-depleted cells showed an increase in cell death marker including cleaved PARP and cleaved caspase9 (Supplementary Fig. S4b) followed by a huge increase in apoptosis compared to that in the control cells (Supplementary Fig. S4c). Taken together, these results imply that USP47-mediated IK stabilization contributes to the ATM-mediated DDR through proper *ATM* pre-mRNA splicing (Fig. 8e).

## Discussion

The spliceosomal B-specific protein IK and SMU1, which are known to be involved in B to B^act^ transition, interact not only with each other but also with the U2 snRNP protein, SF3B3, and the RNA helicase, Brr2, to link the two proteins. Furthermore, knockdown of IK or SMU1 in human cells causes significant alternative splicing of many genes involved in cell death and survival^33^. During this work, Keiper et al.^8^ reported that IK and SMU1 play a critical role in the excision of short introns. In other words, introns retained after the knockdown of IK or SMU1 were predominantly shorter than 200 nt, whereas the majority of introns found in human cells are much longer than 200 nt. In the case of introns having a long distance between the 5-SS and BS, the U2 domain is flexible enough to move toward Brr2. However, in the case of short introns (~56 nt or less), it is hard to move because of a structural constraint. To overcome this problem, IK and SMU1 extend the U2/Brr2 bridge. In this study, we showed that depletion of IK impaired the proper splicing of ATM pre-mRNA. Although ATM has many introns, only intron 1 was not spliced in absence of IK because it has a short length. This result clearly supports the fact that IK plays a critical role in excision of short introns.

In addition to the splicing function, several studies have shown that IK and SMU1 are also involved in genomic stability^34–37^. One previous study showed that the loss of SMU1 function de-represses the DNA replication and over-activates the ATR-dependent replication checkpoint^36^. Although IK depletion leads to decrease in SMU1 level, loss of IK did not phosphorylate CHK1 upon DNA damage. Next, we have examined the level of CHK1 phosphorylation in SMU1-depleted cells. The SMU1 depletion resulted in the phosphorylation of CHK1 upon NCS treatment (Supplementary Fig S5). Thus, it remains to uncover how reciprocal regulation of IK and SMU1 affect DDR independently.

The ATM protein signaling facilitates the repair of DNA damage in an early response to the DSBs^38–42^. The Mre11–Rad50–Nbs1 (MRN) complex autophosphorylates ATM after exposure to DNA damage^43,44^ which subsequently phosphorylates hundreds of substrates, including CHK1, CHK2, H2AX, p53, c-Abl, and FANCD2^45^, thus, leading to DNA repair and cell cycle arrest. In addition to ATM, the protein levels of the MRN complex were also decreased in IK-depleted cells (Supplementary Fig. S6a), but no change in the mRNA levels of Mre11, Rad50, and Nbs1 (Supplementary Fig. S6b). Because Mre11 and Rad50 genes do not have introns shorter than 200 bp, the decrease in protein levels was not because of the splicing failure of these mRNAs. In addition, the IK depletion-lowered ATM is not fully able to phosphorylate Nbs1 when activated by HU, which would weaken the Nbs1 and Mre11 interaction. The resulting Mre11 and Rad50 complex would be dysfunctional. Thus, it is likely that IK is involved in DDR through a proper splicing of ATM. On the other hand, ATM is also involved in DNA damage-induced spliceosome remodeling. The activated ATM reorganizes the distribution of spliceosomes on the pre-mRNA, thereby, causing dissociation of the spliceosomes from the pre-mRNA^27,28^. This spliceosome displacement results in the formation of R-loops at the DNA lesions, which alter 40% of the genome-wide UV-induced alternative splicing (AS) events. Therefore, the loss of the ATM protein in IK-depleted cells may concomitantly cause defects in the ATM-dependent spliceosome mobilization, thus, leading to wide-ranging changes in AS events.

IK/SMU1 is also involved in proper spindle attachment through interaction with MAD1 and causes cell cycle arrest at the mitotic phase. Other studies have demonstrated the connections between pre-mRNA splicing and the cell cycle^46–49^. In general, splicing is inhibited in extracts of mitotic cells, like the other steps in gene expression. Shin and Manley^50^ showed that the SR (rich in serine and arginine) protein, SRp38, represses the splicing during the mitotic phase through mitotic phase-specific dephosphorylation of SRp38. We also observed that IK localized at nuclear speckles and dissociated during mitotic phase (Supplementary Figs. S2 and 5b). Thus, splicing would be inhibited during mitotic phase through some other mechanism and IK released from nuclear speckle might play a role in the proper segregation of chromosomes. Hence, there should be a mechanism to keep IK from dissociation of nuclear speckle with independent functions and we show that USP47 may perform this role at the mitotic phase.

As DUBs modulate the half-life of many cellular proteins related to the signaling pathway, tumor suppression, tumorigenesis, immune response, and DDR, defects in DUBs induce various pathophysiological processes in the cell^51,52^. Among the DUBs, many are associated with DDR. USP14 regulates the IR-induced NHEJ DNA repair^53^. USP49 deubiqutinates p53 to prevent its degradation, and thus, USP49-depleted colon tumors have low levels of p53, which makes them more susceptible to DNA damage-inducers^54^. USP1 removes the mono-ubiquitination of PCNA, and thus UV-induced USP1 cleavage enables monoubiquitinated PCNA to accumulate and to activate translesion synthesis (TLS)^55^; as a result, USP1 knock-out mice are genetically unstable and hypersensitive to DNA damage. DNA damage-induced phosphorylation of USP10 moves it into the nucleus, allowing it to stabilize p53^56^ and USP28 stabilizes Chk2 and 53BP1 in response to DNA damage^57^. USP47 is identified as a major deubiquitinase responsible for the stabilization of DNA polymerase β, which plays a critical role in DNA base excision repair^58^. In this study, we found that USP47 deubiquitinates the spliceosomal factor IK and associates with DDR through the complete splicing of ATM by IK stabilization.

Moreover, recent studies have revealed that pre-mRNA splicing depends on ubiquitination and deubiquitination cycles of the spliceosomal component. For example, the tri-snRNP proteins, PRP3 and PRP31, are reported to regulate the spliceosome through ubiquitination and deubiquitination^17,59–61^. First, the ubiquitin ligase, PRP19, containing the U-box spliceosomal protein, ubiquitinates PRP3 and PRP31, and then the ubiquitinated PRP3 and PRP31 complex binds PRP8 and stabilizes the tri-snRNP complex. The ubiquitinated PRP3 and PRP31 are deubiquitinated by the ubiquitin-specific proteases, USP4 and USP15, respectively. It facilitates the ejection of U4 proteins from the spliceosome during maturation of its active site and progresses the splicing cycle. Ubiquitination plays a role as a mediator of protein–protein interaction in the above example and also functions in protein destabilization as for IK. For the proper spatio-temporal recruitment of IK into the spliceosomes, the stability of IK should be continuously regulated by the USP47 deubiquitinase. It needs to be investigated as to which E3 ligase ubiquitinates IK under a specific condition. In addition, the possibility that the ubiquitinated IK affects the modification of other spliceosomal complexes like PRP3 and PRP31 cannot be ruled out.

In summary, we found that IK depletion impairs the proper splicing of *ATM* pre-mRNA at exons 1–2, which results in ATM deficiency followed by chromosome fragmentation and that USP47 directly binds to IK and deubiquitinates it leading to its stabilization. Thus, we provide the evidence that USP47 is indirectly involved in DDR through the complete splicing of *ATM* pre-mRNA by stabilization of the spliceosomal protein IK.

## Supplementary information

Supplementary Fig. S1

Supplementary Fig. S2

Supplementary Fig. S3

Supplementary Fig. S4

Supplementary Fig. S5

Supplementary Fig. S6

Supplementary Table S1

Supplementary figure legend
