# Supplementary figures and images for: Deubiquitinase USP47-stabilized splicing factor IK regulates the splicing of ATM pre-mRNA
Source: Cell Death Discov. 2020 May 4;6:34. doi: 10.1038/s41420-020-0268-1 (PMC7198525; doi:10.1038/s41420-020-0268-1)

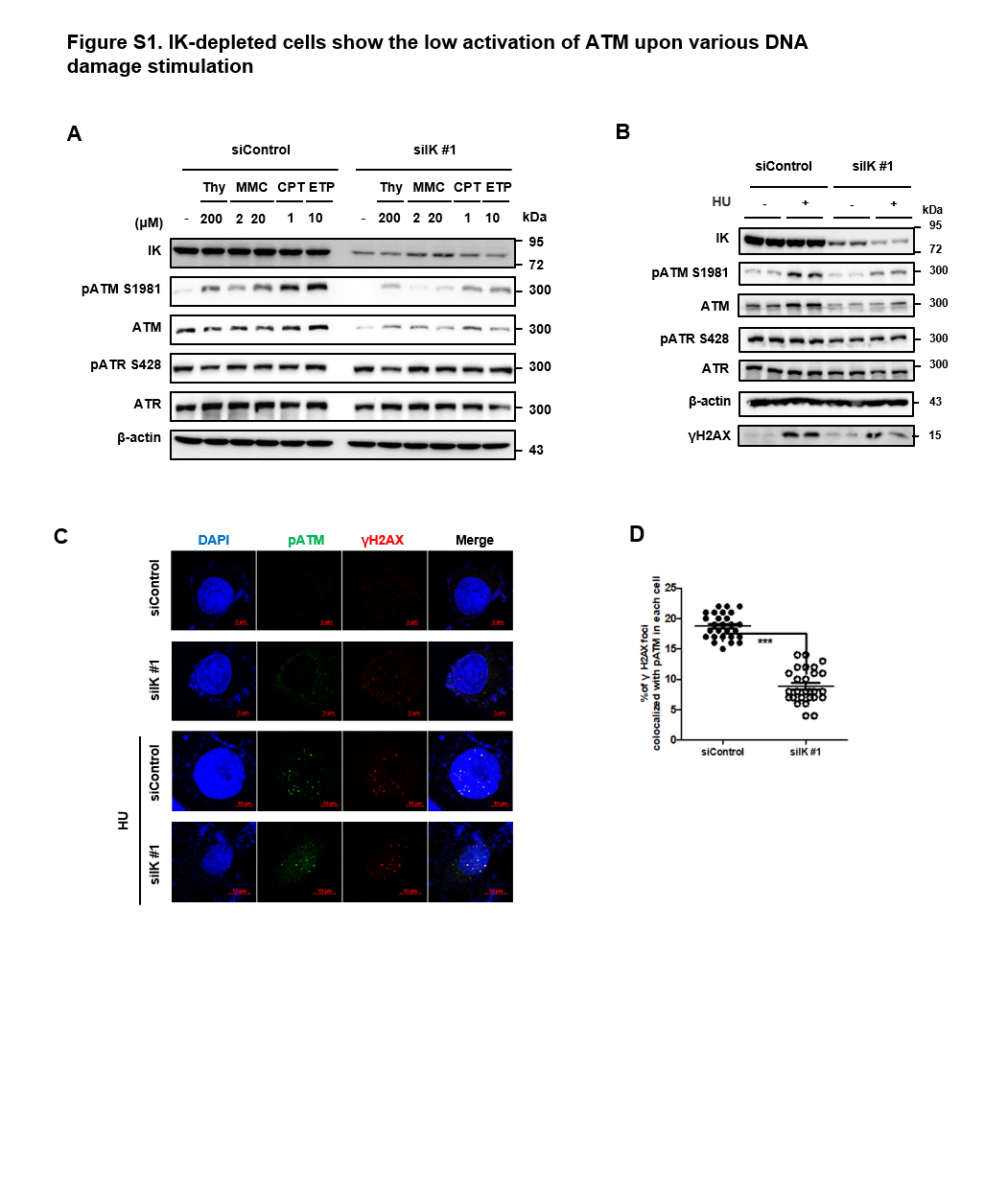

Supplement: Supplementary file 1 — Supplementary Fig. S1 [file 41420_2020_268_MOESM1_ESM.tif]

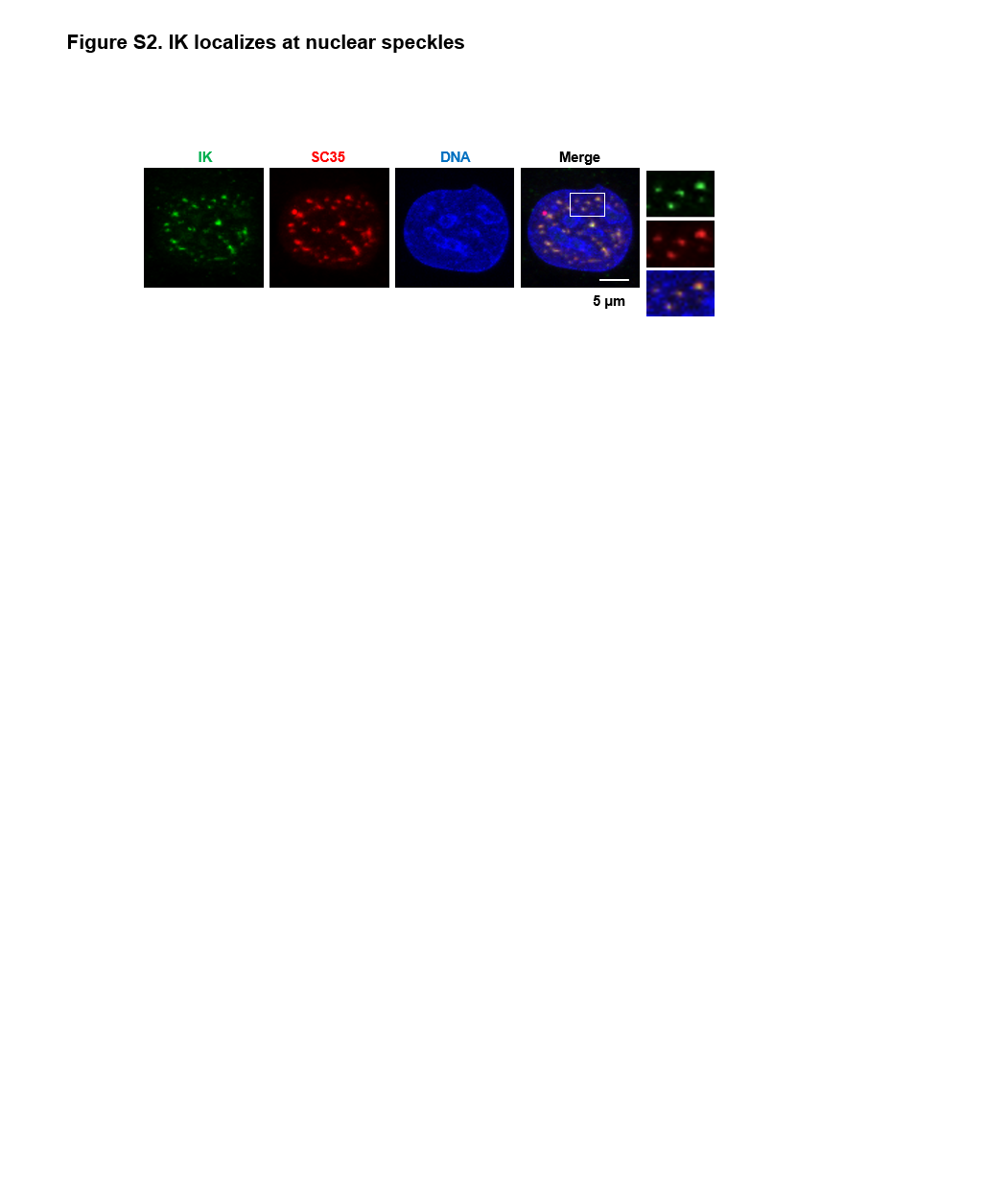

Supplement: Supplementary file 2 — Supplementary Fig. S2 [file 41420_2020_268_MOESM2_ESM.tif]

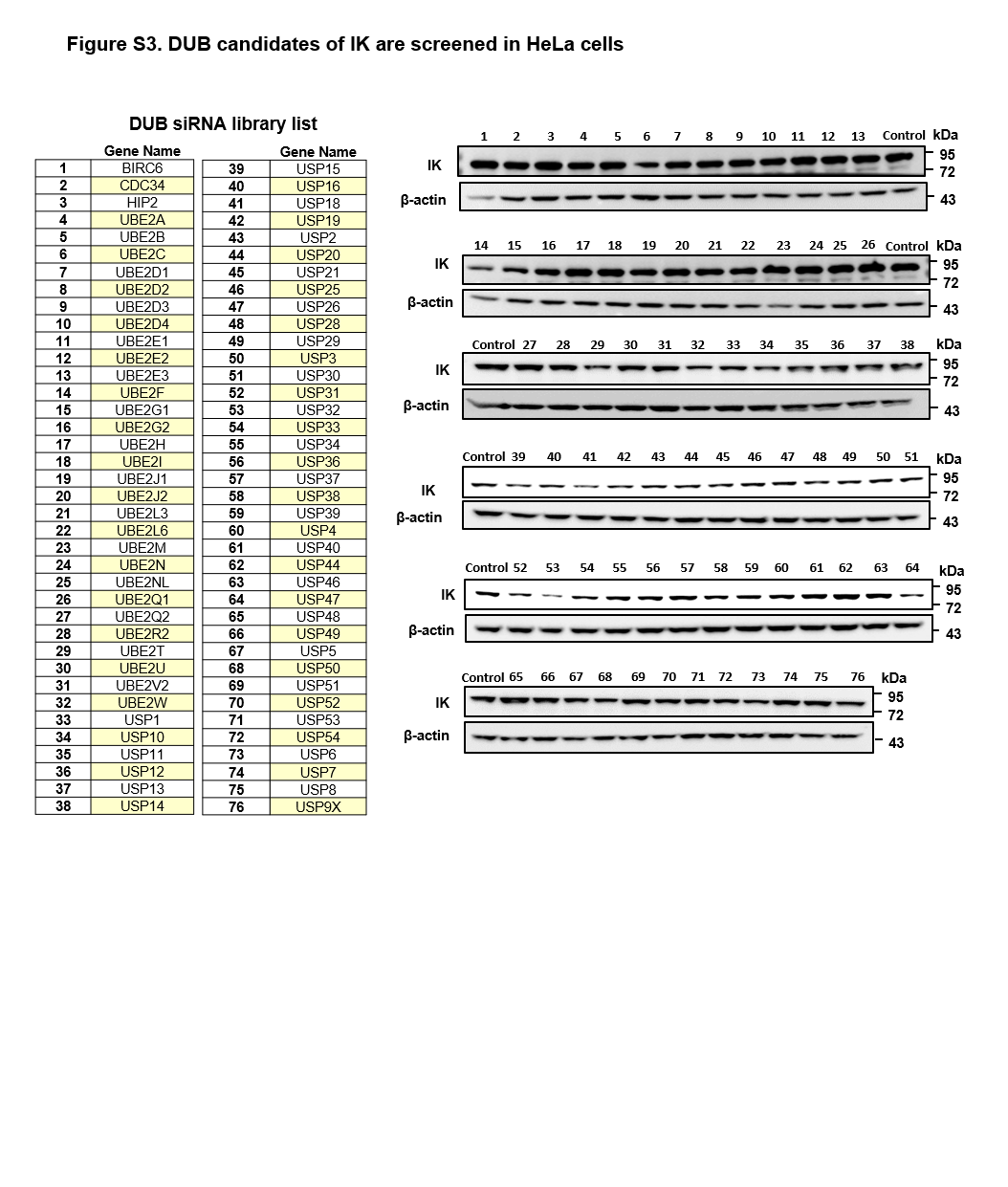

Supplement: Supplementary file 3 — Supplementary Fig. S3 [file 41420_2020_268_MOESM3_ESM.tif]

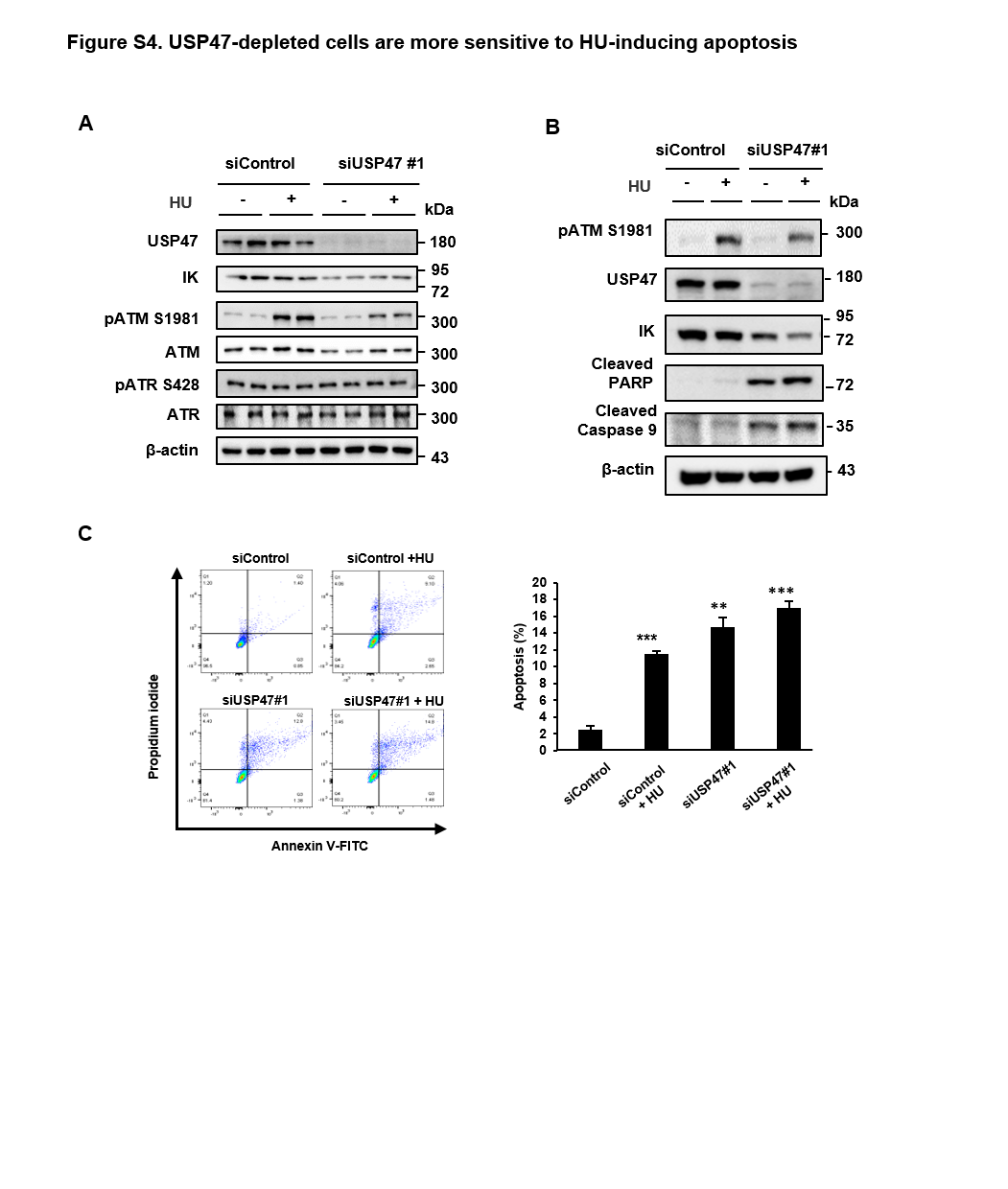

Supplement: Supplementary file 4 — Supplementary Fig. S4 [file 41420_2020_268_MOESM4_ESM.tif]

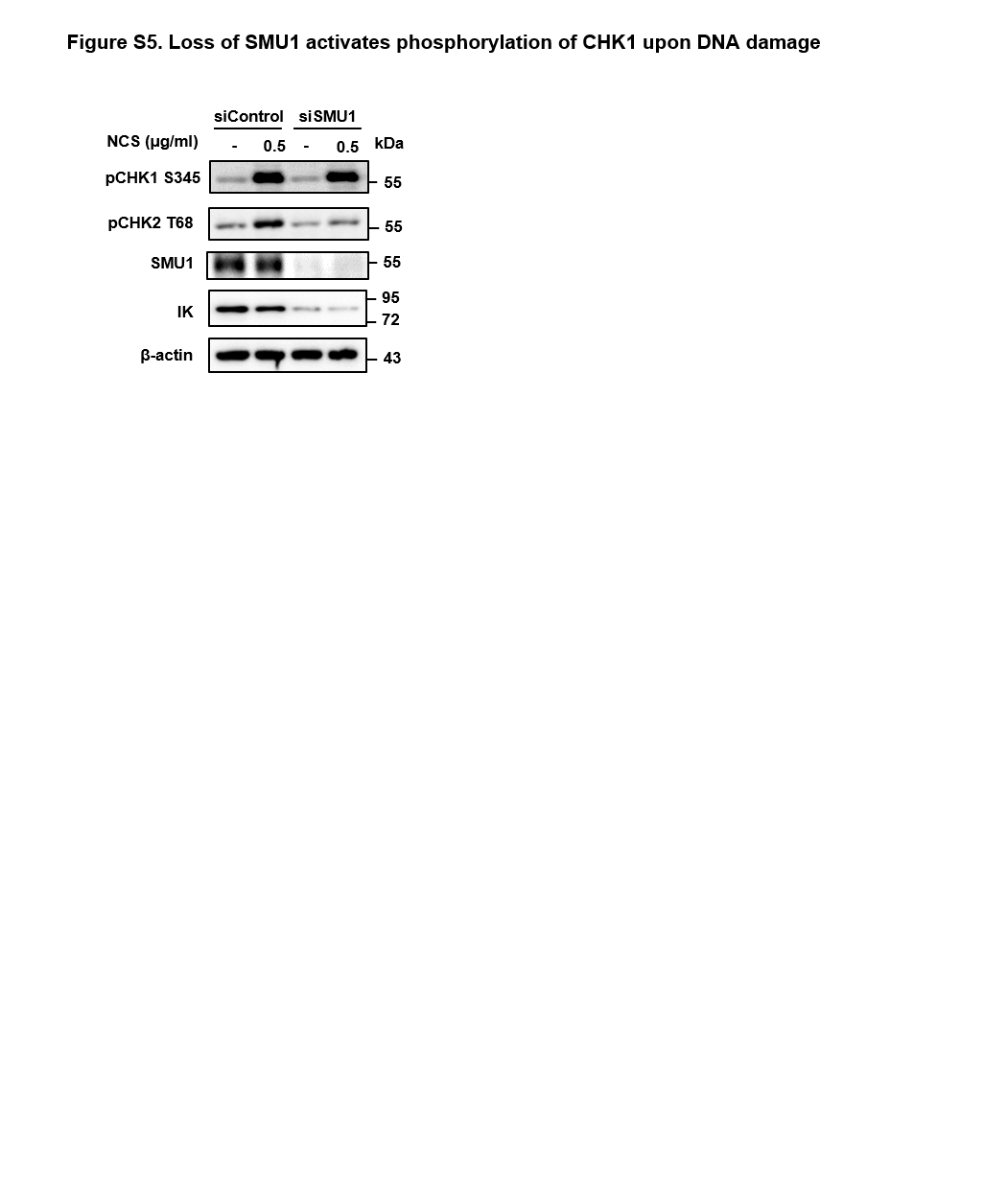

Supplement: Supplementary file 5 — Supplementary Fig. S5 [file 41420_2020_268_MOESM5_ESM.tif]

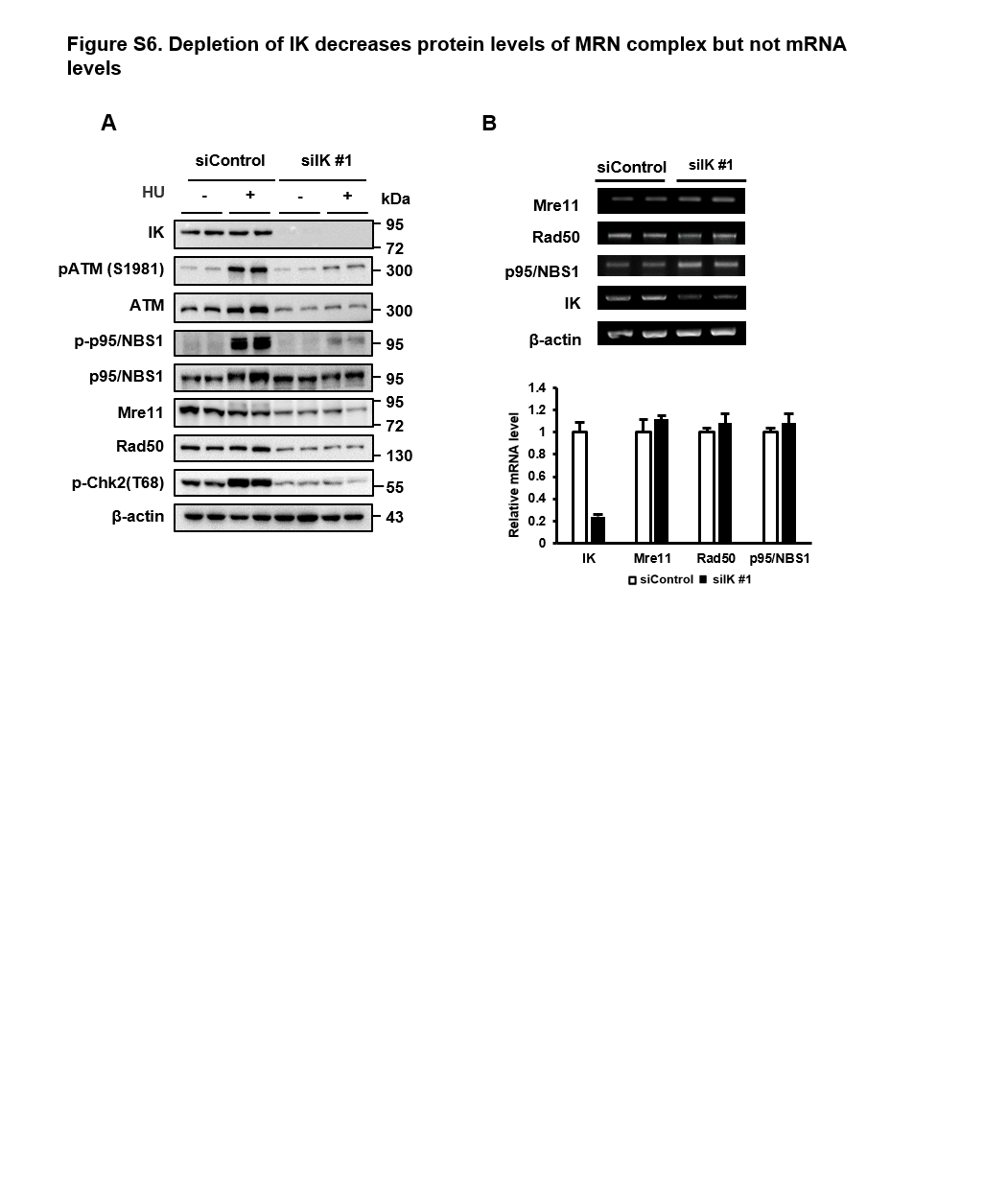

Supplement: Supplementary file 6 — Supplementary Fig. S6 [file 41420_2020_268_MOESM6_ESM.tif]

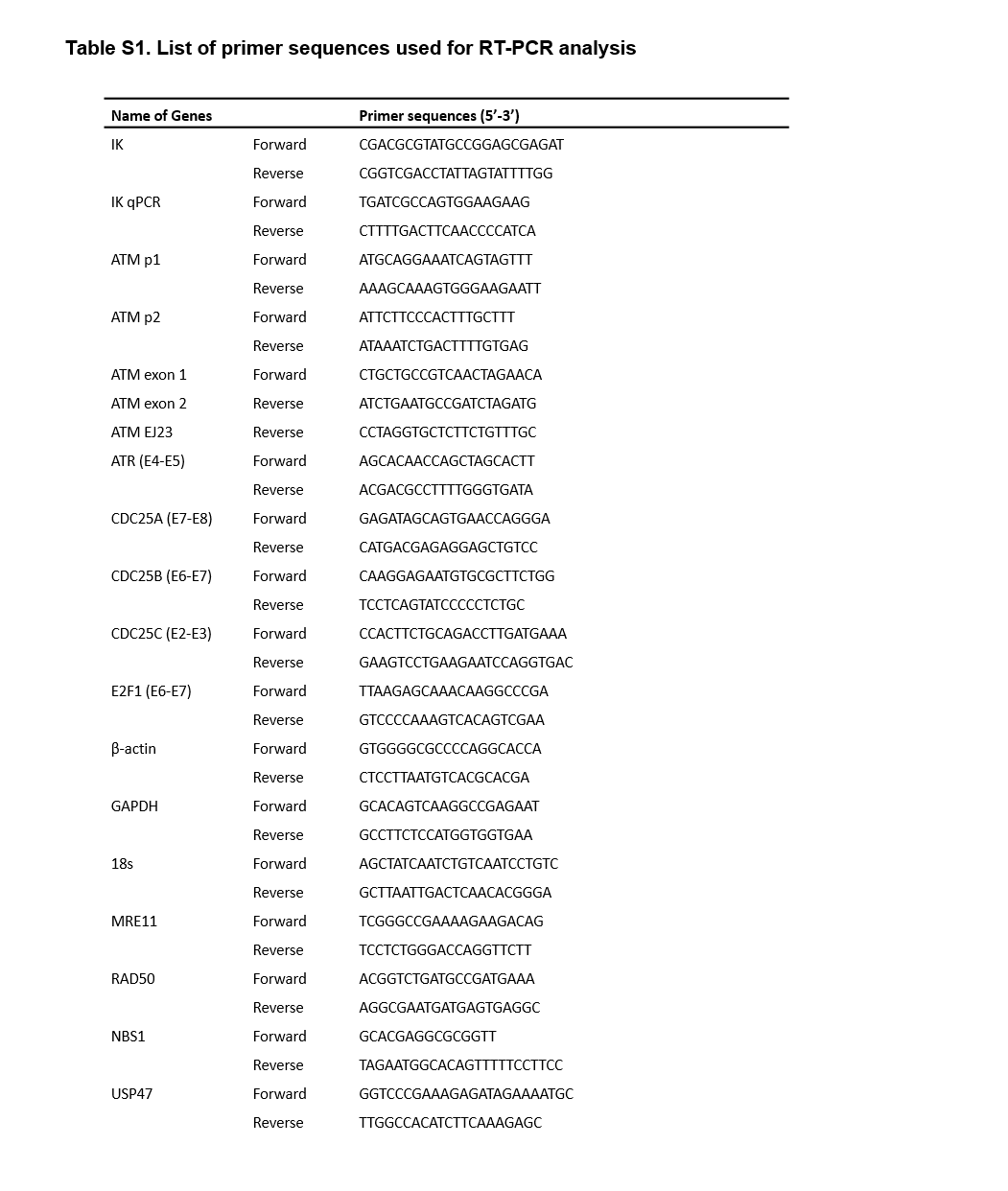

Supplement: Supplementary file 7 — Supplementary Table S1 [file 41420_2020_268_MOESM7_ESM.tif]
