# Supplementary material for: Deubiquitinase USP47-stabilized splicing factor IK regulates the splicing of ATM pre-mRNA
Source: Cell Death Discov. 2020 May 4;6:34. doi: 10.1038/s41420-020-0268-1 (PMC7198525; doi:10.1038/s41420-020-0268-1)
Supplement: Supplementary file 8 — Supplementary figure legend [file 41420_2020_268_MOESM8_ESM.docx]

**Supplementary Figure S1. IK-depleted cells show the low activation of ATM upon various DNA damage stimulation**

**(A)** Cells transfected with siIK #1 for 24 h were treated with the indicated DNA-damaging drugs for an additional 24 h, and the levels of IK, pATM S1981, ATM, pATR S428, and ATR were examined. **(B)** Cells transfected with siIK #1 for 24 h were treated with the hydoxyurea (HU) for an additional 24 h, and the levels of IK, pATM S1981, ATM, pATR S428, ATR, and γH2AX were examined. **(C)** Cells transfected with siIK #1 for 24 h were treated with HU at 2 mM for an additional 24 h and stained with DAPI, anti-pATM, and anti-γH2AX antibody. **(D)** Quantification of the staining in (C), showing the percentage of γH2AX foci co-localized with pATM foci in each nucleus in siControl and siIK #1 after HU treatment. ***p < 0.001.

**Supplementary Figure S2**. **IK localizes at nuclear speckles**

Cells stained with anti-IK or anti-SC-35 antibodies examined via confocal laser microscopy.

**Supplementary Figure S3**. **DUB candidates of IK are screened in HeLa cells.**

HeLa cells were transfected for 48 h with individual 76 DUB siRNAs at 20 nM. Next, the level of endogenous IK was measured by immunoblotting.

**Supplementary Figure S4. USP47-depleted cells are more sensitive to HU-inducing apoptosis**

**(A)** Cells transfected with siUSP47 #1 for 24 h were treated with hydoxyurea (HU) for an additional 24 h, and the levels of IK, pATM S1981, ATM, pATR S428, and ATR were examined. **(B)** Cells transfected with siUSP47 #1 for 24 h were treated with HU for an additional 24 h, and the levels of IK, pATM S1981, PARP, cleaved caspase 3, and cleaved caspase 9 were examined. **(C)** Cells transfected with siUSP47 #1 for 24 h were treated with HU for an additional 24 h, and apoptosis was evaluated using an Annexin V-FITC apoptosis detection kit and flow cytometry. Representative images are obtained using FlowJo software, and the relative percentage of apoptosis was graphed. **p < 0.01, ***p < 0.001

**Supplementary Figure S5. Loss of SMU1 activates phosphorylation of CHK1 upon DNA damage**

Cells transfected with siSMU1 for 47 h were treated with NCS for an additional 1 h, and the levels of pCHK1 S345 and pCHK2 T68 were examined.

**Supplementary Figure S6**. **Depletion of IK decreases protein levels of MRN complex but not mRNA levels**

**(A)** HeLa cells transfected with siIK #1 for 24 h were treated with the hydoxyurea (HU) at 2mM for an additional 24 h, and the levels of IK, pATM S1981, ATM, p-p95, p95, Mre11, Rad50, and pChk2 T68 were examined.

**(B)** HeLa cells were transfected with siIK #1 for 48 h and mRNA levels of Mre11, Rad50, and p95 were analyzed through RT-PCR.

**Table S1. List of primer sequences used for RT-PCR analysis**
